# Experimental Investigation of the Influence of Milling Conditions on Residual Stress in the Surface Layer of an Aerospace Aluminum Alloy

**DOI:** 10.3390/ma18040811

**Published:** 2025-02-12

**Authors:** Magdalena Zawada-Michałowska, Kamil Anasiewicz, Jarosław Korpysa, Paweł Pieśko

**Affiliations:** Department of Production Engineering, Lublin University of Technology, ul. Nadbystrzycka 38D, 20-618 Lublin, Poland; k.anasiewicz@pollub.pl (K.A.); j.korpysa@pollub.pl (J.K.); p.piesko@pollub.pl (P.P.)

**Keywords:** residual stress, aluminum alloy, machining conditions, surface layer, milling

## Abstract

In this study, the correlations between milling conditions—namely, the cutting tool feed direction relative to the rolling direction, the milling type, the coolant application, as well as the cutting speed—and the surface residual stress of a selected aluminum alloy (2024 T351) were investigated. Determining the type and magnitude of residual stress is of paramount importance as this stress is among the primary causes of post-machining strain of thin-walled components. On the basis of the experimental results, it was found that all factors analyzed significantly affect the residual stress state. Specifically, milling in the parallel direction induces lower residual tensile stress compared to milling in the perpendicular direction. Analogously, up-milling yields lower tensile residual stress than down-milling, and flood cooling leads to lower tensile residual stress than MQL. It was clearly confirmed that as cutting speed increases, tensile residual stress also increases, but only up to a certain threshold; once the high-speed cutting regime is reached, tensile residual stress begins to decrease. Consequently, the proper selection of milling parameters is a crucial consideration for optimizing machining processes and minimizing machining-induced residual stress.

## 1. Introduction

Machining is one of the most commonly employed technological processes in mechanical engineering [1]. Often, it constitutes the final stage of production. It enables the manufacturing of parts with diverse shapes over a wide dimensional range, characterized by both high dimensional and shape accuracy, as well as good surface quality. During this process, the cutting tool acts on the workpiece material by removing a layer of it, which, among other effects, leads to changes in the structure and mechanical properties of the material’s surface layer. One of the most significant outcomes of these changes is residual stress, i.e., internal stress present in the material even after the applied force has been removed. During machining, the material near the tool’s cutting edge undergoes non-uniform plastic deformation due to external factors, primarily the cutting force and temperature, which ultimately induces residual stress [2]. Residual stress in the surface layer substantially affects strength, plasticity, and surface integrity, as well as plays a key role in the durability and wear resistance of structural components. It is also cited as one of the leading cause of fatigue cracking, deformation, and corrosion [3].

The most widely recognized models describing the generation of residual stress in a material during machining are as follows [4,5]:

- Mechanical model—assumes that residual stress is generated solely by the cutting force; compressive stress occurs in the surface layer, while tensile stress is present in the deeper zone;
- Thermal model—assumes that residual stress arises exclusively due to temperature; tensile stress forms in the surface layer, whereas compressive stress occurs in the deeper zone.

It should be noted that throughout this complex process, all of these factors influence the residual stress state, although their effects may vary in intensity. The mechanical model (cutting force) is typical of chip-forming machining, whereas the thermal model (temperature) primarily corresponds to abrasive machining and high-performance cutting [4]. Moreover, structural changes can occur during machining, causing volume changes in the material [6].

Residual stress can exert both positive and negative influences on the performance of a manufactured component. Generally, compressive stress (denoted as “−”) in the surface layer yields beneficial effects by enhancing the material’s resistance to creep and fatigue corrosion, increasing strength, and reducing the risk of microcrack formation. By contrast, tensile stress (denoted as “+”) produces the opposite effect. It promotes fatigue cracking, weakens strength, and leads to intergranular corrosion. Depending on specific requirements, the goal is to obtain residual stress of a particular sign.

It is important to note that residual stress arises throughout the entire manufacturing cycle of a part and may also develop during its subsequent operation [7]. The authors of [8,9,10] divided residual stress into two types. The first type pertains to initial residual stress remaining in the material before machining (e.g., formed during the production of the semi-finished product); whereas the second type is machining-induced residual stress, generated during machining. Hence, it should be assumed that the final value and sign of residual stress depend on all operations within the technological process.

It is also crucial to mention the machining of thin-walled components and the significant role of residual stress in this area [11]. This stress leads to post-machining strain. It is a particularly critical issue for parts that require strict tolerances to maintain functionality and reliability. From a safety perspective, this is especially vital in the aviation industry [12]. It is estimated that aerospace companies, including the Boeing Company, spend billions of dollars annually to compensate for strain in machined parts [13,14]. Researchers around the globe are investigating ways to minimize post-machining strain in thin-walled components [15]. However, this problem is highly complex, and achieving a comprehensive solution remains challenging due to the multitude of factors that influence this strain [16,17]. Based on an analysis of residual stress generation models in machining, especially the possibility of concurrent mechanical and thermal influences, it can be inferred that an appropriate selection of machining conditions can significantly reduce strain in thin-walled parts. Solving this issue is important from both scientific and practical standpoints [18].

### 1.1. Machining-Induced Residual Stress of Aluminum Alloys

Given the importance of machining-induced residual stress, researchers worldwide focus on this topic, conducting experimental studies, numerical simulations, and analytical modeling [19]. Table 1 presents a summary of selected studies on the machining-induced residual stress of aluminum alloys.

### 1.2. Residual Stress Relaxation

An important aspect of residual stress is its relaxation, i.e., the spontaneous reduction of stress over time. Understanding the mechanisms of residual stress stabilization and reduction is crucial for ensuring the durability, strength, and reliability of components in various industrial sectors, such as aerospace, automotive, and the broader mechanical engineering industry. One of the most widespread and effective methods of residual stress relaxation is heat treatment. This process involves heating the material to a specific temperature, maintaining it at that temperature for a suitable duration, and then allowing it to cool slowly. The primary form of heat treatment employed for this purpose is stress-relief annealing. Other residual stress relaxation methods are increasingly being developed. These include the use of microwaves (as a supplement to heat treatment), mechanical techniques (e.g., shot peening, which introduces controlled compressive stresses that counteract tensile stresses), and ultrasonic treatment (which facilitates localized dislocation movement). Another approach is natural aging [30].

A review of the relevant literature on aluminum alloys reveals multiple strategies for residual stress relaxation, as reflected in various studies. Zhang et al. [31] reported the relaxation of residual stress in the 7050 aluminum alloy due to aging under both elastic and plastic loading. They conducted a series of tests, including hardness and strength measurements, and elucidated the stress relaxation aging mechanism via electron back-scatter diffraction tests and transmission electron microscopy. It was observed that, regardless of the loading mode, the alloy exhibits a typical two-stage relaxation behavior: an initial rapid decrease in residual stress, followed by a more stable relaxation phase. Song et al. [32] also focused on stress relaxation in the 2219 aluminum alloy under different temperatures, initial stresses, and durations, underscoring the importance of this phenomenon for large integral aerospace components. Their research centered on long-term stress relaxation aging, involving metallographic observations of dislocations and phase precipitates, as well as hardness and strength measurements. Zaroog et al. [33] investigated residual stress relaxation following shot peening in the 2024 T351 alloy. They applied various shot-peening intensities and conducted cyclic tests under two load levels for different numbers of cycles. Residual stress was measured by X-ray diffraction. Yang et al. [34] performed simulation and experimental studies on residual stress relaxation in the 2A14 aluminum alloy during solution treatment and aging. Gao et al. [35] examined the 7075 aluminum alloy subjected to heat treatment, thermovibratory treatment, and vibratory treatment, highlighting the significant impact of these processes on residual stress reduction, with the thermovibratory approach proving most effective. Repplinger et al. [36] investigated stress relaxation under static or cyclic loading at a constant strain level in two 6xxx aluminum alloys, specifically EN AW 6061 T6 and EN AW 6082 T6. Lin et al. [37] utilized annealing for the residual stress relaxation of the 5052H32 aluminum alloy, finding that residual stress decreases rapidly in the initial phase and then remains constant as the annealing time increases at the tested temperatures. Song et al. [38] presented a thermal stress relief method focusing on the distribution of residual stress and conducting additional analyses of mechanical properties, hardness, dislocations, and phase precipitates. Godlewski et al. [39] investigated the effect of aging on residual stress in 319-type aluminum alloy under various temperatures and aging times, observing an increase in residual stress relaxation with increasing aging temperature. Yang et al. [40] proposed an innovative approach to residual stress relaxation under vibration during the transportation of a thin-walled long stringer made of 7050 T7451 aluminum alloy.

However, all of these are additional operations that prolong the manufacturing process and substantially increase costs; thus, in practice, efforts are made to eliminate them. Research focused on the machining process itself, which can be used to “shaping” residual stress, is therefore of critical importance. Such investigations could shorten overall production time and save both labor and energy. Furthermore, they would eliminate costs associated with heat-treatment furnaces and the storage space required for parts during the aging process.

The aim of this study is to comprehensively assess the correlations between machining conditions and surface residual stress of an aerospace aluminum alloy.

## 2. Materials and Methods

Cuboid samples made of 2024 T351 aluminum alloy were used as research material. The independent variables (i.e., the variable machining conditions) were as follows:

- The technological history of the semi-finished product, namely, the cutting tool feed direction relative to the rolling direction (perpendicular or parallel);
- The milling type (up or down);
- The coolant application (minimum quantity lubrication (MQL) with Mobil VG68 oil or flood cooling with MobilCut 230 emulsion);
- The cutting speed (*v_c_* = 150, 450, 750, and 900 m/min).

**Figure 1:**
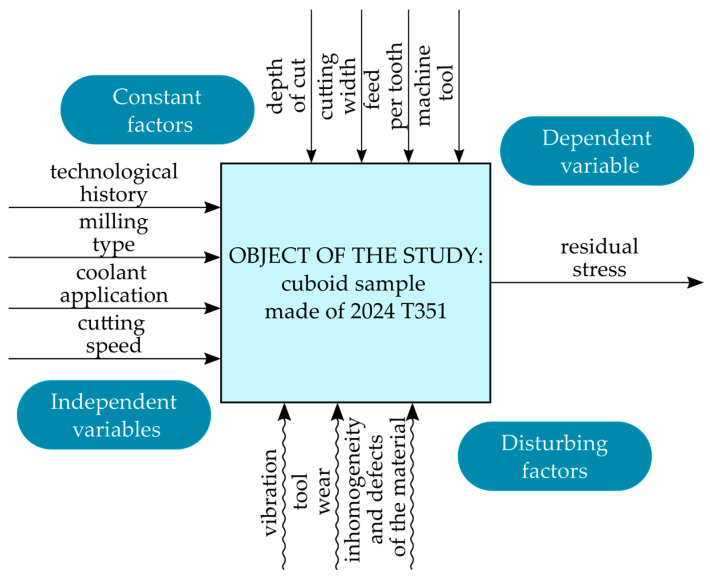
Research plan.

**Figure 2:**
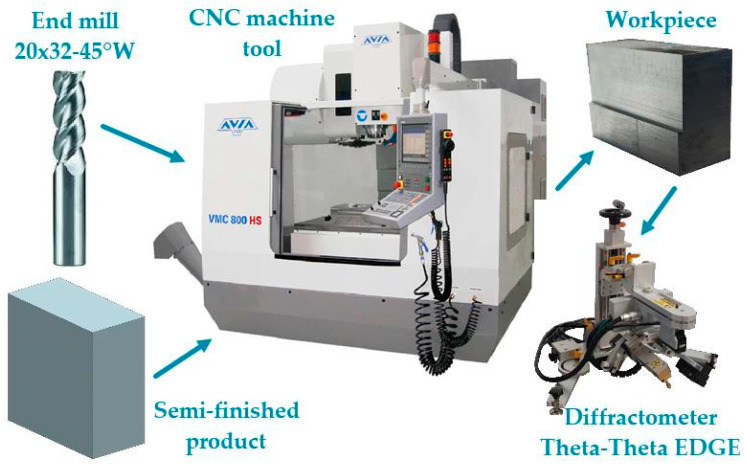
Experimental procedure.

The dependent variable was the residual stress. The constant factors included other technological parameters (depth of cut, feed per tooth, and milling width) and the specific machine tool used. Disturbing factors included vibrations, tool wear, and material inhomogeneity and defects. Figure 1 presents the research plan, while Figure 2 presents the experimental procedure.

Cuboid samples with dimensions of 50 × 25 × 50.8 mm (length × width × height) made of 2024 T351 aluminum alloy were used as research material. The 2024 T351 aluminum alloy is a typical aerospace material whose specification conforms to the AMS4037R standard [41]. The alloy is characterized by high strength, which makes it widely used in aviation—for example, in wing skins. It also offers relatively good machinability, although its corrosion resistance is limited. The semi-finished product was a rolled plate with a thickness of 50.8 mm. Two types of samples were used; i.e., the longer edge of the base was parallel and perpendicular to the rolling direction. This allowed us to determine the influence of material anisotropy on the surface residual stress. Table 2 presents the chemical composition of this alloy, while Table 3 lists selected mechanical properties.

Milling operations were performed on an Avia VMC 800HS vertical machining center (Fabryka Obrabiarek Precyzyjnych AVIA S.A., Warsaw, Poland). A coated solid carbide (VHM) end mill, custom manufactured by FENES (Fabryka Narzędzi Skrawających ‘FENES’ S.A., Siedlce, Poland) under the designation 20x32-45°W, was used. Table 4 presents the technical parameters of the FENES 20x32-45°W milling tool.

Milling was performed in a single tool pass. The following technological parameters were kept constant:

- Depth of cut, *a_p_* = 30 mm;
- Width of cut, *a_e_* = 2 mm;
- Feed per tooth, *f_z_* = 0.1 mm/tooth.

A key stage of the research involved non-destructive surface residual stress measurements carried out using a GNR Theta-Theta EDGE X-ray diffractometer (G.N.R. S.r.l, Agrate Conturbia, Italy). Measurements were taken in five equally spaced locations on each sample. A 4 W lamp with a Cr anode, a V filter, and a 1 mm collimator was used. The measurements followed the sin^2^*ψ* method, using nine offset angles *ψ* in the range of ±35°. Acquisition time was 30 s. X-ray elastic constants were 2, 2, 2, i.e., 2 (h), 2 (k), and 2 (l). The measurements were repeated five times for one variant. The analysis focused on a uniaxial stress state.

## 3. Results

**Figure 3:**
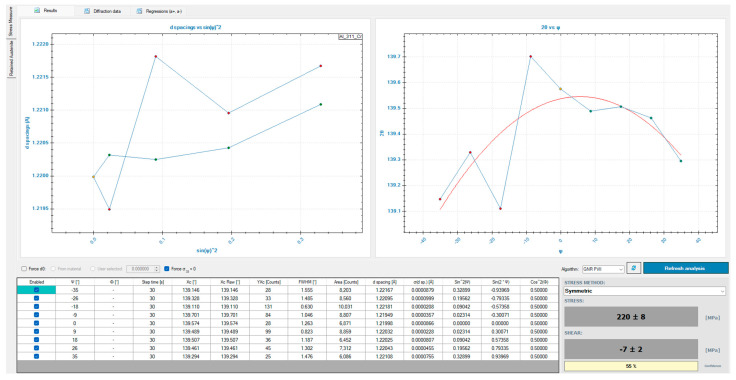
Example of the single measurement result of surface residual stress (*v_c_* = 750 m/min, up-milling, MQL, parallel feed direction relative to rolling direction).

The research findings were presented in a graphical/quantitative format using bar charts, allowing for the analysis of the influence of individual variables, i.e., technological history, milling type, coolant application, and cutting speed, on the residual stress. In each case, the residual stress was plotted as a function of the cutting speed. This approach provided detailed information on the influence of these variables on “shaping” residual stress. Figure 3 presents the single measurement result of surface residual stress for *v_c_* = 750 m/min, up-milling, MQL, and parallel feed direction relative to rolling direction. The results analysis did not take into account shear stress.

### 3.1. Technological History

**Figure 4:**
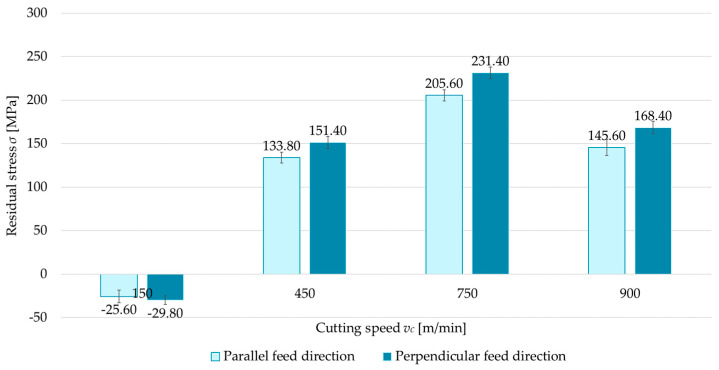
Comparison of residual stress for parallel and perpendicular cutting tool feed directions relative to rolling direction as a function of cutting speed under up-milling and flood cooling.

**Figure 5:**
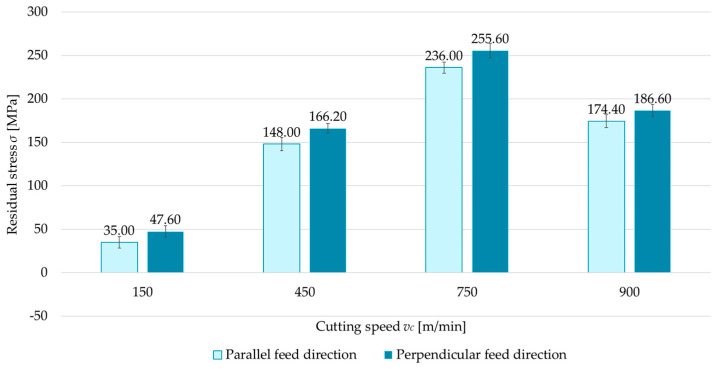
Comparison of residual stress for parallel and perpendicular cutting tool feed directions relative to rolling direction as a function of cutting speed under down-milling and flood cooling.

**Figure 6:**
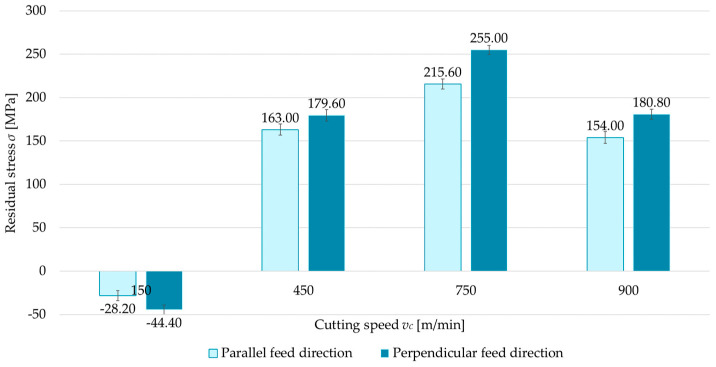
Comparison of residual stress for parallel and perpendicular cutting tool feed directions relative to rolling direction as a function of cutting speed under up-milling and MQL.

**Figure 7:**
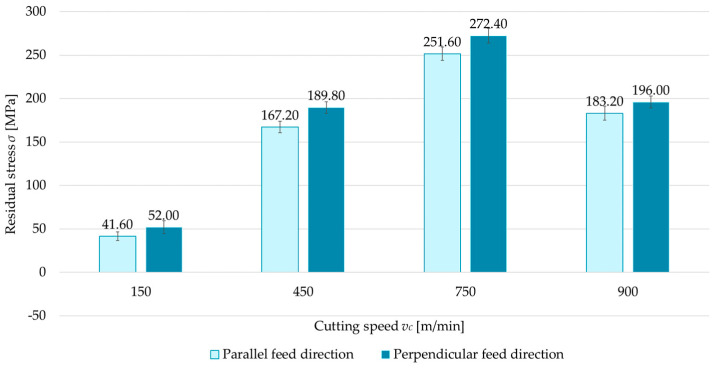
Comparison of residual stress for parallel and perpendicular cutting tool feed directions relative to rolling direction as a function of cutting speed under down-milling and MQL.

The first variable investigated was the technological history—that is, the feed direction of the cutting tool relative to the rolling direction. Two cases were considered: parallel and perpendicular milling directions with respect to the rolling direction. Figure 4, Figure 5, Figure 6 and Figure 7 compare surface residual stress for each feed direction (parallel and perpendicular) in four configurations (up/down-milling and flood cooling/MQL). Analysis of the results indicated that lower residual stress was obtained when milling in the parallel direction relative to the rolling direction. Additionally, it was observed that compressive stress occurred at a cutting speed of *v_c_* = 150 m/min for up-milling. This behavior was confirmed for both the perpendicular and parallel milling directions with respect to the rolling direction, whereas all other variants exhibited tensile stress. The residual stress for the parallel direction was 6.5–15.5% lower than for the perpendicular direction. At *v_c_* = 150 m/min, regardless of configuration, the differences ranged from 14.1% to 36.5% (relative to perpendicular milling).

### 3.2. Milling Type

**Figure 8:**
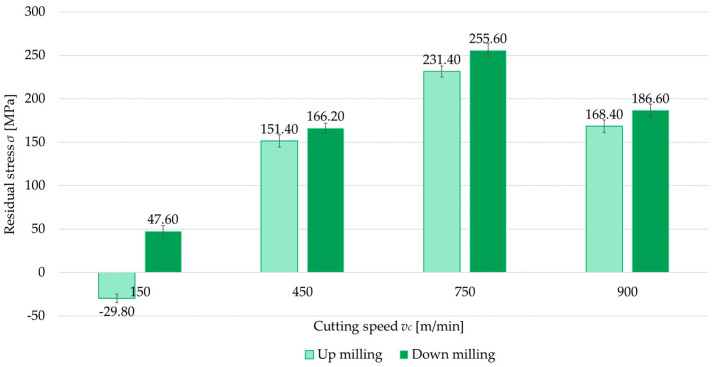
Comparison of residual stress for up and down-milling as a function of cutting speed under perpendicular feed direction relative to rolling direction and flood cooling.

**Figure 9:**
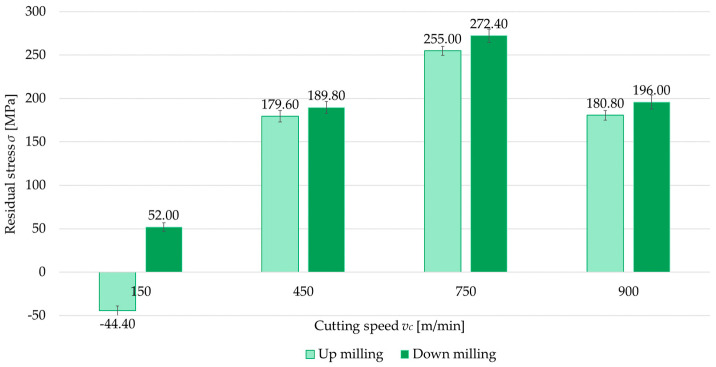
Comparison of residual stress for up and down-milling as a function of cutting speed under perpendicular feed direction relative to rolling direction and MQL.

**Figure 10:**
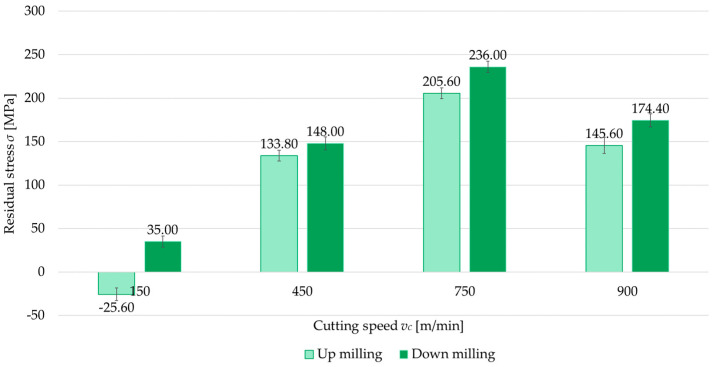
Comparison of residual stress for up and down-milling as a function of cutting speed under parallel feed direction relative to rolling direction and flood cooling.

**Figure 11:**
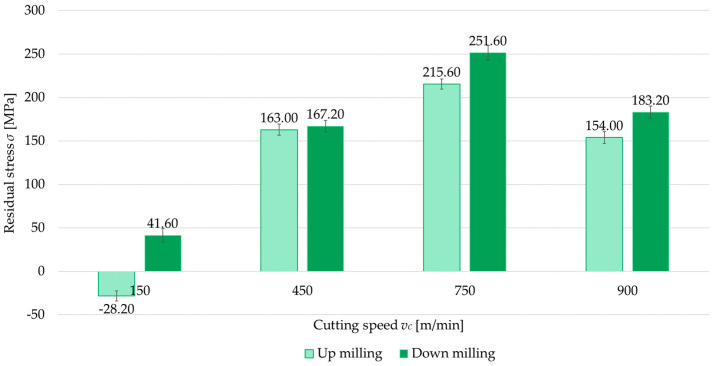
Comparison of residual stress for up and down-milling as a function of cutting speed under parallel feed direction relative to rolling direction and MQL.

The second independent variable examined was the milling type (up- and down-milling). Figure 8, Figure 9, Figure 10 and Figure 11 present a comparison of surface residual stress as a function of milling type across various studied (parallel/perpendicular cutting tool feed direction relative to rolling direction and flood cooling/MQL). On the basis of the results, lower residual stress was obtained under up-milling. At a cutting speed of *v_c_* = 150 m/min, it was observed that up-milling resulted in compressive (negative) stress, whereas down-milling induced tensile (positive) stress. This outcome is linked to the dominance of the cutting force (mechanical model) in the former case and the dominance of the temperature (thermal model) in the latter. For *v_c_* = 150 m/min, the residual stress under up-milling was, on average, 170% lower compared to down-milling. At other cutting speeds, only tensile stress was observed, with the difference ranging from 5.5% to 15.5% (relative to down-milling). In just one variant (MQL with parallel feed direction relative to rolling direction), the difference between stress was 2.5%.

### 3.3. Coolant Application

**Figure 12:**
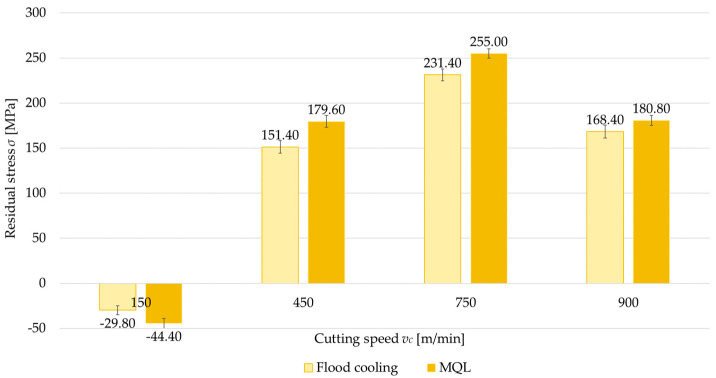
Comparison of residual stress for flood cooling and MQL as a function of cutting speed under perpendicular feed direction relative to rolling direction and up-milling.

**Figure 13:**
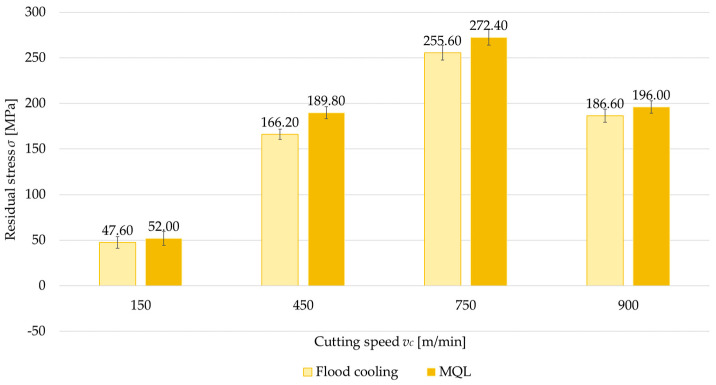
Comparison of residual stress for flood cooling and MQL as a function of cutting speed under perpendicular feed direction relative to rolling direction and down-milling.

**Figure 14:**
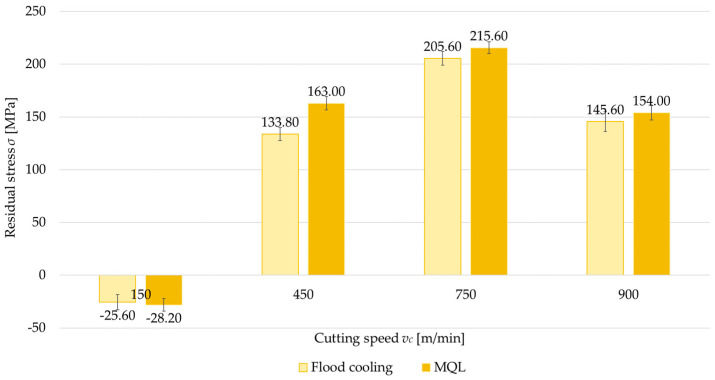
Comparison of residual stress for flood cooling and MQL as a function of cutting speed under parallel feed direction relative to rolling direction and up-milling.

**Figure 15:**
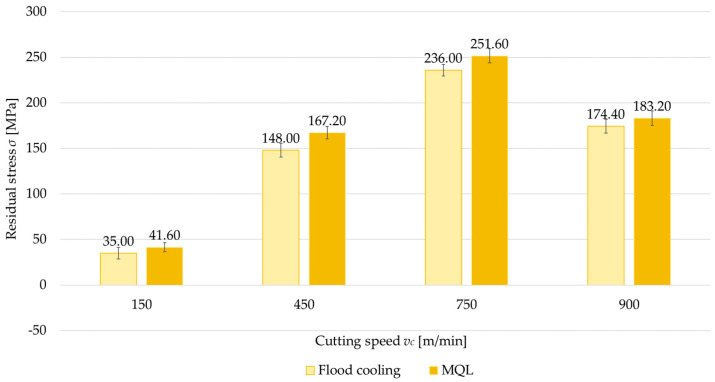
Comparison of residual stress for flood cooling and MQL as a function of cutting speed under parallel feed direction relative to rolling direction and down-milling.

Another factor analyzed was the application of cooling, considered in two variants: flood cooling and MQL. Figure 12, Figure 13, Figure 14 and Figure 15 show a comparison of surface residual stress for flood cooling and MQL across the investigated configurations. Based on the results, it was found that lower residual stress was obtained with flood cooling. For up-milling (Figure 12 and Figure 14) at a cutting speed of *v_c_* = 150 m/min, compressive stress was observed (regardless of flood cooling or MQL). In contrast, for down-milling (Figure 13 and Figure 15) at *v_c_* = 150 m/min, tensile stress was observed (again regardless of flood cooling or MQL). At all other cutting speeds, tensile stress was noticed. These findings confirm the observations reported in Section 3.1 and Section 3.2. The residual stress under flood cooling was approximately 5% to 18% lower than that under MQL. At *v_c_* = 150 m/min, when combining a perpendicular tool feed direction relative to the rolling direction with up-milling, this difference exceeded 30% compared to MQL.

### 3.4. Cutting Speed

**Figure 16:**
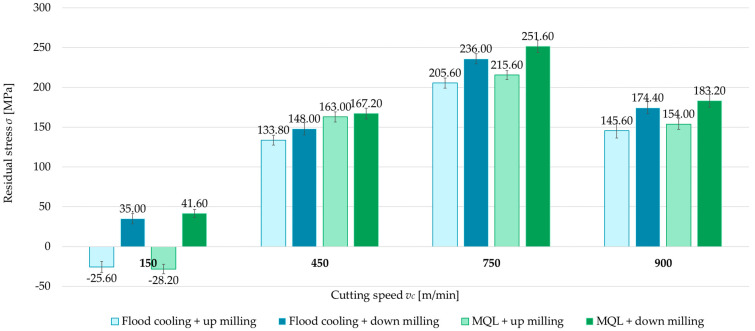
Residual stress as a function of cutting speed with different milling combinations and parallel feed direction of the cutting tool with respect to the rolling direction.

**Figure 17:**
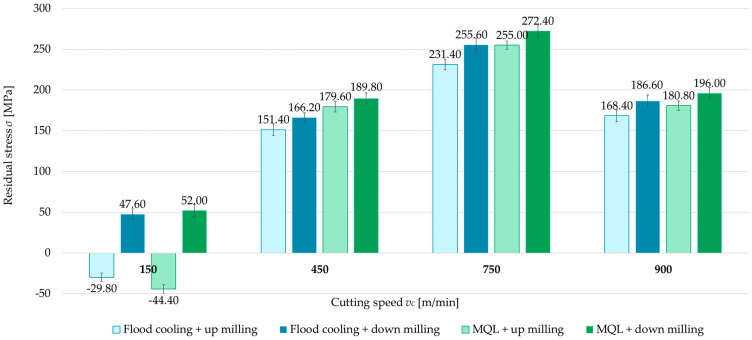
Residual stress as a function of cutting speed with different milling combinations and perpendicular feed direction of the cutting tool relative to the rolling direction.

The last variable analyzed is cutting speed (*v_c_* = 150, 450, 750, and 900 m/min). Figure 16 and Figure 17 show comparisons of the surface residual stress as a function of cutting speed with different milling combinations and the parallel and perpendicular feed direction of the cutting tool relative to the rolling direction. From the results, it can be observed that as the cutting speed increases, the residual stress increases, but only up to a certain speed. The residual stress at *v_c_* = 750 m/min reached a maximum, while a decrease was recorded at *v_c_* = 900 m/min. In most cases, tensile residual stress was obtained. The exception is up-milling at *v_c_* = 150 m/min. In this variant, compressive residual stress was obtained. The difference between the maximum residual stress (*v_c_* = 750 m/min) and the minimum stress (*v_c_* = 150 m/min) averaged 115% for both parallel and perpendicular rolling directions. It was also observed that the residual stress at *v_c_* = 900 m/min and *v_c_* = 450 m/min was comparable.

### 3.5. ANOVA

The obtained test results were also subjected to statistical analysis based on a four-way ANOVA (Table 5). The analysis confirmed that all four variable factors had a significant effect on the value of residual stress. Due to the large effect of cutting speed on the test results, it showed significant interactions with the other variables. On the contrary, no interactions were shown between the other variables, indicating that they affected the machining effect in a similar way.

**Figure 18:**
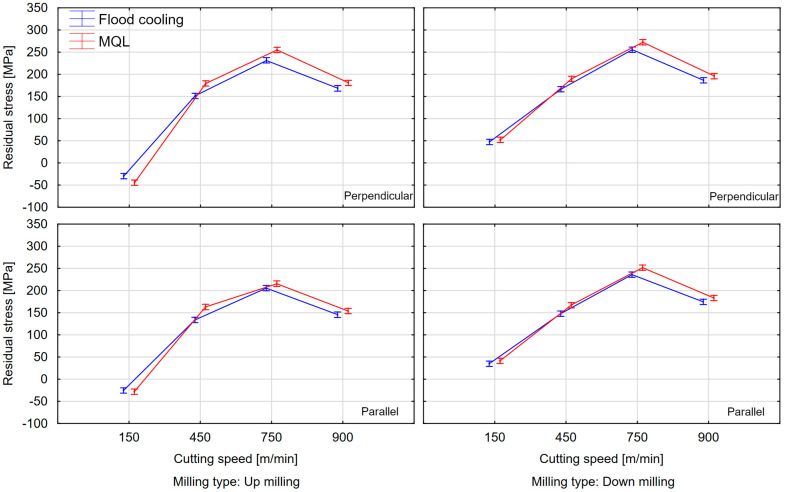
Interaction plots for ANOVA.

This is also confirmed by the graphs shown in Figure 18. It can clearly be seen that the main influence on the stress value was the cutting speed, while the trend of changes were similar regardless of the change in feed direction, milling type, and coolant application.

## 4. Discussion

This study aimed to assess the correlations between machining conditions and residual stress in the surface layer of an aerospace aluminum alloy (2024 T351). Four machining variables were investigated: technological history, milling type, coolant application, and cutting speed.

In the first stage, attention was paid to the technological history, which is understood as the cutting tool feed direction (either parallel or perpendicular) relative to the rolling direction. Investigations into the influence of milling orientation with respect to the rolling direction indicated that parallel milling produces lower tensile residual stress in comparison to perpendicular milling. This outcome likely arises from the fact that parallel milling proceeds along the grain direction (i.e. the microstructure developed during rolling), leading to fewer disruptions in the crystal structure. Furthermore, the machining-induced residual stress is more evenly distributed on the surface, reducing its accumulation. The reduced interaction of the tool with structural inhomogeneity (e.g., segregated bands) in the rolled material also helps to decrease tensile stress. By contrast, perpendicular milling involves working against the grain orientation, causing the tool to “cut across” the structural bands and increasing the intensity of interactions in the cutting zone. The greater number of microstructural disruptions leads to higher tensile residual stress. That the material anisotropy affects the surface residual stress is an important conclusion of this study. The differences in machining effects depending on the cutting tool feed direction relative to the rolling direction are a subject of considerations for many scientists; e.g., Cerutti et al. [42] emphasized that before machining, which is usually the last stage of production process, the workpiece undergoes many manufacturing steps involving uneven plastic deformations that are a source of residual stress, and this is crucial for the quality of the manufactured parts.

Next, a focus was placed on the milling type, specifically comparing up-milling to down-milling. The results showed that up-milling yields lower tensile residual stress than down-milling. The main reason for this phenomenon is the difference in how the cutting force acts on the material. During up-milling, increased plastic deformation occurs in the cutting zone, resulting in higher compressive stress, which affects both the magnitude and the sign of the overall residual stress. This explanation is supported by the observation that at a cutting speed of *v_c_* = 150 m/min, up-milling produces compressive (negative) stress, whereas at the same cutting speed, down-milling produces tensile (positive) stress.

Another variable examined was the coolant application, considered in two variants: flood cooling and MQL. The results confirmed that flood cooling during machining reduces tensile residual stress compared with MQL. This effect is primarily related to a decrease in cutting temperature. The higher temperature generated during MQL causes thermal expansion of the material and microstructural changes that can lead to thermal stress. However, flood cooling effectively lowers the cutting temperature, thereby limiting thermal expansion and minimizing structural changes in the surface layer. Additionally, reduced friction is an important factor. Flood cooling lowers the friction between the cutting edge and the workpiece, diminishing the severity of plastic deformation in the surface layer.

In the final stage, attention was focused on the effect of cutting speed on residual stress. Four cutting speeds were analyzed, including one corresponding to high-speed cutting. The findings demonstrate that the cutting speed significantly influences residual stress through both heat generation and process mechanics. At *v_c_* = 150 m/min, the cutting process generates relatively low heat, exerting minimal impact on the microstructure of the material. Increasing the cutting speed leads to more intense heat generation and microstructural transformations, such as dynamic recrystallization and grain growth in the surface layer, which tend to promote tensile stress. At *v_c_* = 900 m/min, a reduction in cutting force was also observed, consistent with the findings of Magdalena Zawada-Michałowska et al. in [5], likely due to decreased cutting resistance. The results indicate that there is an optimal range of cutting speeds to minimize residual stress, reflecting a compromise between heat generation and the degree of mechanical deformation.

It should be noted that many researchers focus primarily on the influence of technological parameters on machining-induced residual stress (e.g., refs. [24] or [43]). The novelty effect in our research is the comprehensive assessment of four independent variables—cutting tool feed orientation relative to rolling direction, milling type, coolant application, as well as cutting speed—and their effects on the magnitude and sign of the surface machining-induced residual stress. The results allow for milling process optimalization, assuming the minimization of residual stress as the optimization criterion. The appropriate selection of machining conditions can effectively “shape” the state of the residual stress, which is of crucial importance for the quality and durability of components.

It should be also noted that residual stress is the main factor causing distortion in thin-walled components, so future investigations should focus on parts with thin walls, as was described by Zawada-Michałowska et al. [44], which fits into the scientific field of the authors of this article. Further research should also take into account the residual stress distribution in the material, as in [25].

## 5. Conclusions

Based on the results obtained, the following conclusions can be drawn:

1. The state of residual stress (sign and value) is influenced by all analyzed factors, i.e., cutting tool feed direction relative to material rolling direction, milling type, coolant application, and cutting speed;
2. By optimizing the machining conditions, it is possible to effectively “shape” the residual stress in the surface layer, which has a direct impact on the quality and durability of the manufactured parts;
3. Up-milling with flood cooling, machining in a direction parallel to the rolling direction, and the optimization of the cutting speed lead to a reduction in the surface residual stress in the material;
4. Residual stress is strongly related to the mechanics of plastic and thermal deformation, as well as to the microstructure of the material;
5. In the future, it will be necessary to extend our research into the geometry corresponding to thin-walled structures and to verify our results in terms of the possibility of minimizing post-machining strain.

The results of this study are relevant to industries where component durability and resistance to fatigue damage are crucial. Reducing residual stress, especially tensile stress, increases the reliability and operational safety of elements.

## Figures and Tables

**Table 1:** Summary of selected papers on machining-induced residual stress of aluminum alloys.

Material	Machining Conditions	Applications	Reference
2219 aluminum alloy	*Experimental study* Square cavity milling; Solid carbide end milling cutter: *d* = 8 mm; *λ_s_* = 55°; *z* = 2; Study of 20 variants of technological parameters: *a_p_* = 0.1–1 mm; *a_e_* = 0.5–2.5 mm; *f_z_* = 0.06–0.09 mm/tooth, *n* = 4000–12,000 rpm; A method for measuring self-stress: X-ray diffractometer.	The depth of cut was found to be the most important parameter affecting the distribution of machining-induced residual stress. It is recommended to use small values of depth of cut to obtain compressive residual stress. Increasing the depth of cut results in tensile residual stress. Rotational speed, feed rate, and milling width have little effect on the distribution of this stress. A change from compressive residual stress to tensile stress was observed with the removal of successive layers of material.	[20]
2024 T351, 7075 T651 aluminum alloys	*Experimental study* Testing of four variants of technological parameters: *a_e_* = 4 and 8 mm; *f_z_* = 0.05 and 0.2 mm/tooth; *v_c_* = 400 and 900 m/min; A method for measuring self-stress: X-ray diffractometer.	It was found that an increase in cutting speed increases the value of residual stress, and compressive residual stress changes to tensile stress.	[21]
7075 T6511 aluminum alloy	*Experimental study* End milling cutter: *d* = 16 mm; *z* = 3; Testing of three variants of technological parameters: *f_z_* = 0.05–0.08 mm/tooth; *v_c_* = 251–500 m/min; *a_e_* = 11 mm; Wet cutting; Stress measurement method: layer removal method and strain gauge.	It was found that increasing the cutting speed to the high-speed machining range reduces the residual stress in the material, while increasing the feed per tooth increases the residual stress. It was observed that machining-induced residual stress improves fatigue life in the low-cycle regime.	[22]
2A12 aluminum alloy	*Experimental study* Carbide end milling cutter: *d* = 12 mm; *z* = 4; *λ_s_* = 30°; Study of nine variants of technological parameters: *f_z_* = 0.05 mm/tooth; *n* = 2000–6000 rpm; *a_p_* = 1–2 mm; *a_e_* = 6–10 mm; Tool path: single-way milling, reciprocating milling, S-shaped milling; Method of stress measurement: hole-drilling measurements (with strain gauge rosette).	The optimal combination of technological parameters was presented so that the residual stress and surface roughness could be minimized (based on gray correlation analysis).	[23]
AA7050 T7451 aluminum alloy	*Experimental–simulation study* Down-milling; Cemented carbide end milling cutter: *d* = 12 mm; *λ_s_* = 45°; *z* = 3; Study of four variants of technological parameters: *f_z_* = 0.04–0.2 mm/tooth; *v_c_* = 200 and 450 m/min; Method of self-stress measurement: hole-drilling measurements (with a rosette type A strain gage); FEM: ABAQUS.	It was found that increasing the feed per tooth results in an increase in the depth of residual stress and a shift in the depth of maximum compressive residual stress deep into the material. In the case of cutting speeds, no significant changes were observed as higher cutting speeds need to be tested.	[24]
7075 T7451 aluminum alloy	*Experimental–simulation study* Milling cutter: *d* = 20 mm; *γ* = 15°; *α* = 10°; End milling: *v_c_* = 125.6, 376.8, and 628 m/min; *f_z_* = 0.01, 0.025, and 0.05 mm/tooth; *a_p_* = 0.5, 1, and 1.5 mm; *a_e_* = 10, 15, and 20 mm; Side milling: *v_c_* = 125.6, 376.8, and 628 m/min; *f_z_* = 0.01, 0.025, and 0.05 mm/tooth; *a_p_* = 10, 15, and 20 mm; *a_e_* = 0.5, 1, and 1.5 mm; Wet cutting; A method for measuring self-stress: X-ray diffraction; Simulation: genetic algorithm and BP neural network.	It was found that the depth of the residual stress is up to 0.12 mm from the surface and their distribution has a “spoon” shape.	[25]
2024 T3 aluminum alloy	*Theoretical–simulation–experimental study* Ball-end milling cutter: *d* = 4 mm; *z* = 2; Study of nine variants of technological parameters: *n* = 8000–12,000 rpm; *f_z_* = 0.075–0.1 mm/tooth; *a_p_* = 0.15–1 mm; *a_e_* = 0.5–1.5 mm; A method for measuring self-stress: X-ray diffraction; FEM: ABAQUS.	A new empirical model of machining-induced residual stress as the effect of milling force and milling heat is presented. It was found that the cutting force causes both compressive and tensile residual stress, while heat only causes tensile stress (in the surface layer).	[26]
7075 T6 aluminum alloy	*Experimental–simulation study* Solid carbide milling cutters: *d* = 10 mm; *z* = 2; *λ_s_* = 25°; Testing of nine variants of technological parameters: *v_c_* = 188.4 m/min; *f_z_* = 0.05 mm/tooth; *a_p_* = 8 mm; *a_e_* = 0.2–1.2 mm; *i* = 1–3 (number of passes); A method for measuring self-stress: X-ray diffraction; FEM: ABAQUS.	It was found that milling in multiple passes can reduce surface tensile residual stress and the value and depth of compressive residual stress.	[27]
2024 T3 aluminum alloy	*Experimental–simulation study* Down-milling; Carbide milling cutter: *d* = 6 mm; *γ* = 15°; *α* = 2–10°; *λ_s_* = 30°; *z* = 4; Technological parameters: roughing: *v_c_* = 65.97 m/min; *f_z_* = 0.2 mm/tooth; *a_p_* = 0.1–0.5 mm; *a_e_* = 4 mm; finishing: *v_c_* = 65.97 m/min; *f_z_* = 0.1 mm/tooth; *a_p_* = 0.01–0.08 mm; *a_e_* = 4 mm; A method for measuring self-stress: X-ray diffraction; FEM: AdvantEdgeTM-3D.	It was found that it is possible to optimize the distribution of residual stress by selecting an appropriate depth of cut. It has been observed that as the depth of cut decreases (in both roughing and finishing), the surface residual stress decreases.	[28]
7055 T7751 aluminum alloy	*Simulation study* Cemented carbide milling cutter: *γ* = 8–16°; *α* = 2–10°; Study of five variants of technological parameters: *a_p_* = 2–10 mm; *f_z_* = 0.1–0.9 mm/tooth; *v_c_* = 1000–5000 m/min; *a_e_* = 4–12 mm; Simulation: a two-dimensional model of the high-speed milling of aluminum alloy based on the equivalent uniform cutting thickness and the finite element numerical simulation in combination with the Johnson–Cook constitutive equation.	Tensile residual stress increases with increasing milling speed, feed per tooth, and milling depth. An increasing trend was also observed for increasing the milling width and face angle, while a decreasing trend was observed for the relief angle. Optimization of technological parameters and tool geometry (including the study of the angles *α* and *λ*) can minimize residual stresses.	[29]

**Table 2:** Chemical composition of aluminium alloy 2024 T351 (own compilation based on inspection certificate).

Chemical Composition [%]													
Si	Fe	Cu	Mn	Mg	Cr	Ni	Zn	Ti	Ga	V	Other-Each-Max	Other-Total-Max	Al
0.19	0.20	4.40	0.60	1.50	0.01	0.004	0.11	0.03	0.01	0.01	0.05	0.15	The rest

**Table 3:** Selected mechanical properties of aluminium alloy 2024 T351 (own compilation based on inspection certificate).

Mechanical Properties		
Tensile Strength	Yield Strength	Elongation
*R_m_* [MPa]	*R_p_*_0.2_ [MPa]	*A_5_* [%]
428	290	6

**Table 4:** Technical parameters of FENES 20x32-45°W (own compilation based on manufacturer data).

FENES 20x32-45°W					
Cutting Diameter [mm]	Shank Diameter [mm]	Cutting Edge Length [mm]	Helix Angle [°]	Number of Flutes [-]	Coating
20	20	32	45	3	TiAlN

**Table 5:** Four-way ANOVA results.

Variable	*SS*	*df*	*MS*	*F*	*p*
Feed direction (A)	10,384.51	1	10,384.51	224.55	0.00
Milling type (B)	46,751.41	1	46,751.41	1010.91	0.00
Coolant application (C)	6187.66	1	6187.66	133.80	0.00
Cutting speed (D)	1,181,400.87	3	393,800.29	8515.18	0.00
A*B	0.06	1	0.06	0.00	0.97
A*C	11.56	1	11.56	0.25	0.62
A*D	3583.17	3	1194.39	25.83	0.00
B*C	15.01	1	15.01	0.32	0.57
B*D	24,773.07	3	8257.69	178.56	0.00
C*D	3784.52	3	1261.51	27.28	0.00
A*B*C	0.06	1	0.06	0.00	0.97
A*B*D	1967.02	3	655.67	14.18	0.00
A*C*D	271.82	3	90.61	1.96	0.12
B*C*D	620.37	3	206.79	4.47	0.01
A*B*C*D	181.52	3	60.51	1.31	0.27

## Data Availability

The original contributions presented in this study are included in the article. Further inquiries can be directed to the corresponding author.
